# Retrospective Identification of Herpes Simplex 2 Virus-Associated Acute Liver Failure in an Immunocompetent Patient Detected Using Whole Transcriptome Shotgun Sequencing

**DOI:** 10.1155/2017/4630621

**Published:** 2017-12-26

**Authors:** Atsushi Ono, C. Nelson Hayes, Sakura Akamatsu, Michio Imamura, Hiroshi Aikata, Kazuaki Chayama

**Affiliations:** ^1^Department of Gastroenterology and Metabolism, Applied Life Sciences, Institute of Biomedical & Health Science, Hiroshima University, Hiroshima, Japan; ^2^Liver Research Project Center, Hiroshima University, Hiroshima, Japan; ^3^Laboratory for Digestive Diseases, SNP Research Center, The Institute of Physical and Chemical Research (RIKEN), Hiroshima, Japan

## Abstract

Acute liver failure (ALF) is a severe condition in which liver function rapidly deteriorates in individuals without prior history of liver disease. While most cases result from acetaminophen overdose or viral hepatitis, in up to a third of patients, no clear cause can be identified. Liver transplantation has greatly reduced mortality among these patients, but 40% of patients recover without liver transplantation. Therefore, there is an urgent need for rapid determination of the etiology of acute liver failure. In this case report, we present a case of herpes simplex 2 virus- (HSV-) associated ALF in an immunocompetent patient. The patient recovered without LT, but the presence of HSV was not suspected at the time, precluding more effective treatment with acyclovir. To determine the etiology, stored blood samples were analyzed using whole transcriptome shotgun sequencing followed by mapping to a panel of viral reference sequences. The presence of HSV-DNA in blood samples at the time of admission was confirmed using real-time polymerase chain reaction, and, at the time of discharge, HSV-DNA levels had decreased by a factor of 10^6^.* Conclusions.* In ALF cases of undetermined etiology, uncommon causes should be considered, especially those for which an effective treatment is available.

## 1. Introduction

Acute liver failure (ALF), also called fulminant hepatic failure, is a rare but life-threatening condition characterized by sudden deterioration of liver function and associated with coagulopathy (international normalized ratio ≥ 1.5) and hepatic encephalopathy [1, 2]. Mortality among ALF patients is high, often resulting from multiorgan failure and brain stem decompression due to cerebral edema [3]. ALF implies that the patient had no prior history of liver disease or cirrhosis [2], providing few clues to the underlying etiology, and the rapid onset and difficulty of identification at the earliest stages may lead to delays in the start of treatment. Nonspecific treatments have limited effectiveness, and ALF accounts for about 7% of liver transplantations [1]. However, spontaneous recovery is observed in up to 45% of ALF patients, and specific treatments for known etiologies can be effective [3]. Acetaminophen overdose is the most common cause of ALF in the United States and Europe, whereas viral hepatitis is more common in Asia and Africa, but numerous other causes have been reported, including drug-induced liver injury, viral hepatitis, ischemic liver injury, Wilson's disease, and acute presentation of autoimmune hepatitis [4, 5]. Aside from hepatitis viruses A, B, and E, other viruses including parvovirus B19, SEN virus, echovirus 18, and several members of the Herpesviridae (e.g., herpes simplex, herpes zoster, Epstein-Barr, and cytomegalovirus) have been reported to cause ALF in rare cases [6–9]. Optimal treatments can differ widely among these etiologies, but, in at least 15% of cases, the etiology cannot be adequately determined, and treatment choices must made based on incomplete information [3]. However, treatment of ALF could be improved by retrospective analysis of cases of ALF with uncertain etiology to establish the risk factors and clinical presentation of these etiologies to aid in future diagnosis. Improvements in rapid sequencing methodology might soon even make it possible to use real-time benchtop sequencing as a diagnostic tool [10, 11]. In this report, we describe an instructive case in which a more effective treatment could have been selected if less common etiologies had been considered upon admission.

Herpes simplex virus (HSV) infection is an uncommon cause of ALF that typically occurs following reactivation as a result of pregnancy or immunosuppression [13–15]. Therefore, it is rarely identified until after death or liver transplantation (LT) [13]. Here, we present a case of HSV-associated ALF in an immunocompetent patient who recovered without LT. We also suggest the usefulness of next generation RNA sequencing and real-time polymerase chain reaction of stored blood samples for retrospective identification of pathogens causing ALF from stored blood samples.

## 2. Case Presentation

A previously healthy 18-year-old Japanese female initially presented to her local hospital with fever and pharyngodynia. She reported that she had not had sexual intercourse within the previous 6 months. Based on physical findings, including fever and presence of swollen tonsils with white pus-filled spots, as well as laboratory findings that revealed liver dysfunction (AST: 373 IU/L, ALT: 461 IU/L) and inflammatory status (white blood cell count: 18600 *μ*/L, CRP: 5.4 mg/dL), a diagnosis of infectious mononucleosis was made. She was hospitalized, and treatment with 200 mg/day of minocycline and 300 mg/day of hydrocortisone was started. The next day, she exhibited symptoms of right abdominal pain, and computed tomography (CT) scan revealed ascites. The diagnosis of Fitz-Hugh-Curtis Syndrome was suspected, and treatment of 2 g/day of azithromycin was initiated. However, she presented with worsening of liver function. Drug-induced liver injury was suspected, and all antibiotics were discontinued. After withdrawal of the drugs, she presented with coagulopathy and further worsening of liver function tests, wherein she was transferred to our hospital.

On admission to our hospital, the patient's mental status was Glasgow coma scale (GCS) 13 (eye opening: 3; verbal response: 4; best motor response: 6), hepatic encephalopathy grade 2, and her body temperature was 38.1°C. Other vital signs were stable as follows: blood pressure 114/67 mmHg, heart rate 88 bpm, SpO2 99% in room air, and respiratory rate 17/min. No skin abnormalities were identified. A laboratory analysis revealed severe liver dysfunction, coagulopathy, inflammation, thrombocytopenia, and anemia (Table 1).

**Figure 1:**
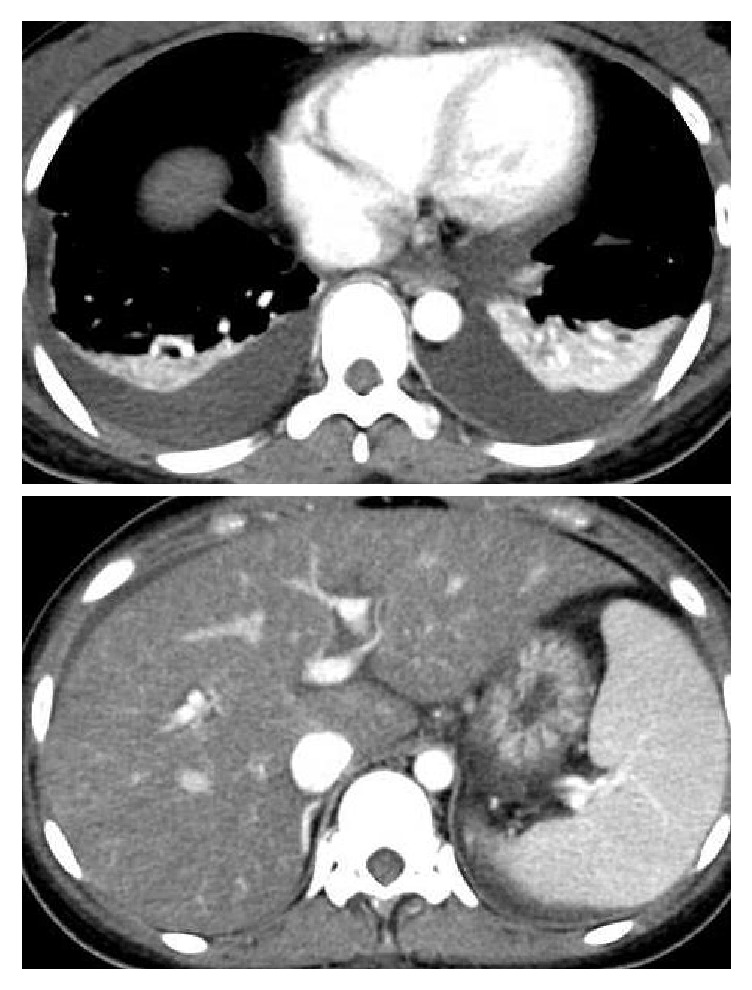
Dynamic contrast-enhanced CT scan.

**Figure 2:**
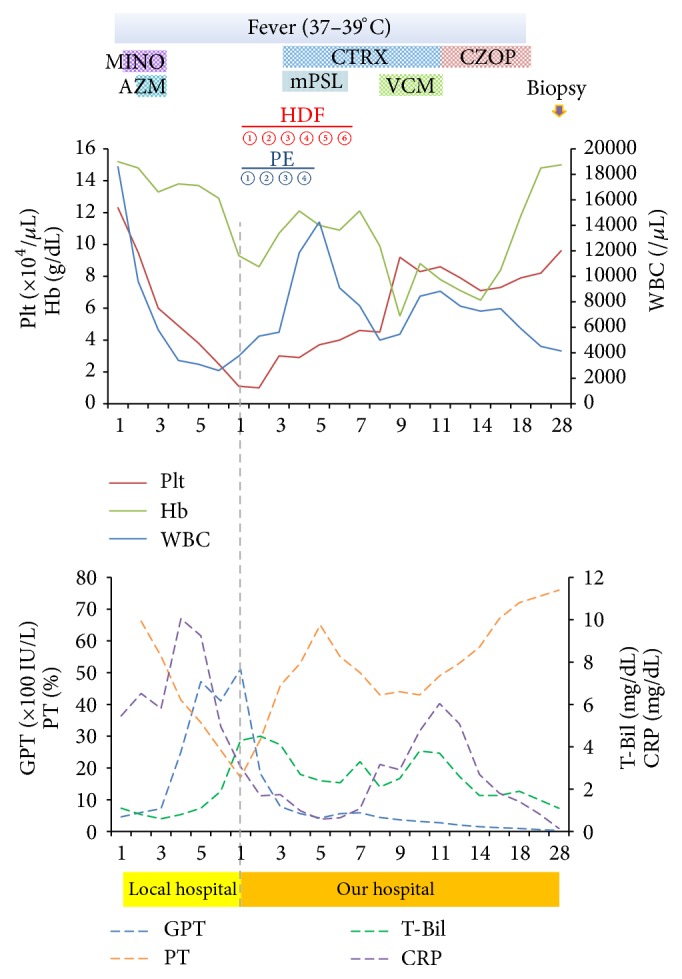
The patient's clinical course. MINO: minocycline, AZM: azithromycin, CTRX: ceftriaxone, VCM: vancomycin, CZOP: cefozopran; mPSL: methylprednisolone sodium succinate.

**Figure 3:**
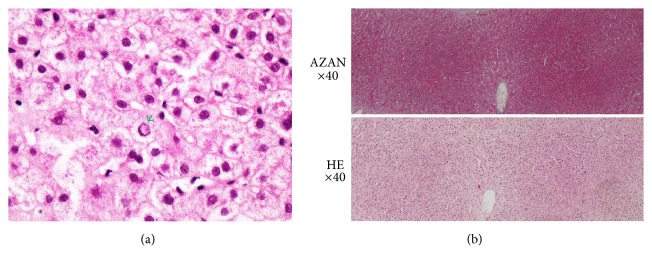
Findings of liver needle biopsy. (a) The arrow indicates a nuclear inclusion body. (b) Low magnification (×40) of a liver fragment with HE and AZAN staining.

An abdominal and chest dynamic contrast-enhanced CT scan revealed periportal collar sign, splenomegaly, and a small amount of ascites and pleural effusion (Figure 1). No obvious hepatic atrophy was observed. The patient was diagnosed with ALF based on criteria published by the Ministry of Health, Labour, and Welfare of Japan [5, 16], although the etiology was unknown (drug-induced ALF was suspected). The patient was treated six times with hemodialysis filtration (HDF) and four times with peritoneal dialysis (Figure 2). Although the origin of the fever was not determined, infectious disease was suspected, and the patient was treated with antibiotics as shown in Figure 2. Liver needle biopsy was performed on day 29. Histopathological findings showed piecemeal necrosis and nuclear inclusion bodies (Figure 3(a)). Neither extensive necrosis nor obvious parenchymal hepatocytes or liver fibrosis was observed. The pathological diagnosis of a fragment of liver tissue indicated periportal hepatocellular loss and regenerative bile duct (Figure 3(b)). Focal necrosis accompanied by lymphocyte infiltration and eosinophilic body is scarcely recognized in the hepatocytes in the lobule. Some of the hepatocytes around the Gleason sheath are accompanied by piecemeal necrosis and show extensive shedding, but the reconstructed image is poor. The appearance of multinucleated hepatocytes is observed at another site in the leaflet. The Gleason sheath is fibrillary in part with mild infiltration of chronic inflammatory cells and mild neutrophil infiltration. Aggregation of regenerated bile ducts was observed. No cholestasis was apparent in hepatocytes or bile ducts. These findings appear to be consistent with acute liver injury.

The patient recovered from ALF and was discharged from the hospital on day 30.

### 2.1. RNA Sequencing

In order to reveal the cause of her liver failure, we retrospectively screened her stored blood samples by RNA sequencing for the presence of viral RNA. The patient provided written informed consent, and the study was performed in accordance with the World Medical Association Declaration of Helsinki and with the approval of the local ethics committee. RNA-Seq, also called whole transcriptome shotgun sequencing, involves isolation of sample RNA, depletion of ribosomal RNA, and synthesis of cDNA, which is then sequenced using next generation sequencing technology. RNA was extracted from stored blood samples, and fragmented RNA was linked with RNA 3′ adapter. Reverse transcription and polymerase chain reaction were performed using single-stranded cDNA templates. Sequencing was performed using the HiSEQ 2500 platform (Illumina, Tokyo, Japan) using paired end reads. Reads were trimmed using Trimmomatic (version 0.36), and quality scores were examined before and after trimming using FastQC (version 0.11.3). Bowtie2 [17] was used to map the RNA reads against 7,195 sequences from the viral RefSeq collection downloaded from the National Center for Biotechnology Information (downloaded on April 17, 2016). All sequence analysis was performed on the Shirokane 3 supercomputer at the University of Tokyo.

### 2.2. Results of RNA Mapping

**Figure 4:**
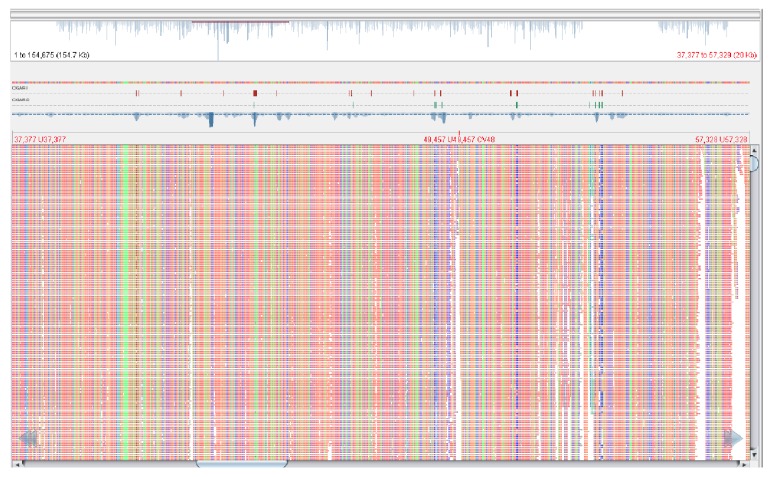
Alignment of patient serum RNA against human herpesvirus 2 reference genome. Reads were aligned using Bowtie2 and displayed using Tablet viewer [12].

Results of Bowtie2 mapping revealed matches against three viral RefSeq sequences: NC_022518.1 (human endogenous retrovirus K113: 164 reads), NC_023677.1 (chimpanzee alpha-1 herpesvirus strain 105650: 264 reads), and NC_001798.2 (human herpesvirus 2 strain HG52: 270,696 reads). After remapping, 1,216,468 out of a total of 139,632,418 reads (0.87%) mapped to the NC_001798.2 human herpesvirus 2 reference genome, overlapping 80% of the 154,657 nt reference sequence with an average coverage depth of 768 and a maximum coverage depth of 6,991 (Figure 4).

### 2.3. Quantification of HSV-DNA

**Figure 5:**
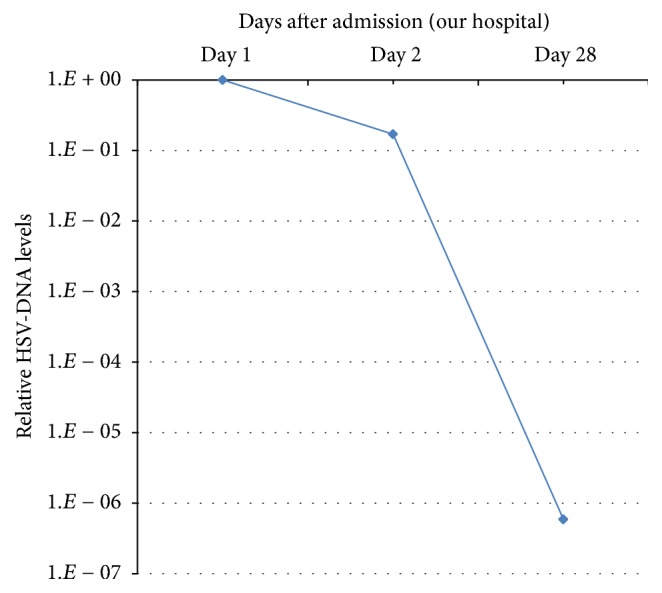
Relative HSV-DNA levels on days 1, 2, and 28. HSV-DNA levels decreased to lower than 10^−6^ from day 1 to day 28.

Based on the results of read mapping, real-time PCR was used to retrospectively quantify relative HSV-DNA levels in stored blood samples. DNA was extracted from 100 *μ*L of serum (day 1 and day 2) and plasma (day 28) using SMI-TEST (Genome Science Laboratories, Tokyo, Japan) according to the manufacturer's instructions and dissolved in 20 *μ*L of distilled water. Real-time PCR analysis was performed using the Mx300P System (Applied Biosystems, Foster City, CA) according to the instructions provided by the manufacturer. We prepared 10 *μ*L of Brilliant III Ultra-Fast SYBR Green QPCR Master Mix with Low ROX (Agilent Technologies, Santa Clara, CA), 100 nM of forward primer: TCAGCCCATCCTCCTTCGGCAGTA (target: Glycoprotein G, Nucleotide position: 138240–138262; derived from GenBank accession number Z86099), 100 nM of reverse primer: CGCGCGGTCCCAGATCGGCA (target: Glycoprotein G, Nucleotide position: 138398–138380), 2 *μ*L of DNA, and 7.6 *μ*L of distilled water. The cycling program was as follows: the sample was heated for 3 minutes at 95°C for denaturing, followed by a PCR cycling program consisting of 40 two-step cycles of 5 seconds at 95°C and 20 seconds at 60°C. RT-PCR analysis confirmed the presence of HSV-DNA in the patient's blood samples at the time of admission and revealed that HSV-DNA levels had decreased by a factor of 10^−6^ between days 1 and 28 (Figure 5), shortly after which the patient was discharged. Acute HSV infection in this patient was also confirmed by a positive test for HSV-IgM antibody (8.6: normal range, <0.8) measured using stored serum samples.

## 3. Discussion

Although acute liver failure can result from several conditions, including viral hepatitis, drug-induced liver injury, and autoimmune hepatitis, in many cases the underlying cause cannot be conclusively determined. However, prognosis for this condition is poor, and failure to identify the cause may prevent selection of an effective treatment [15]. The findings presented here indicate that the patient had an undiagnosed acute HSV infection on admission to our hospital. Acute HSV infection in this patient was also confirmed by the presence of HSV-IgM antibody. HSV-2 is sexually transmitted, resulting in a lifelong, incurable infection and recurrent outbreaks of genital herpes. However, we can only speculate on when and how the patient was infected, and, considering that the patient was underage, it is unclear if she was unaware of the nature of the infection or perhaps reluctant to report it. We acknowledge this as a limitation of our study. Because HSV is an uncommon cause of ALF in nonimmunosuppressed and nonpregnant patients, the diagnosis of HSV infection was not considered during the acute phase in the case we presented. Although she was fortunately able to recover, diagnosis of HSV infection and antiviral treatment should have been initiated as soon as possible. Hence, the patient presented here was an instructive case that cautioned us to acknowledge the need to suspect HSV infection in patients with ALF even when the patient is neither pregnant nor immunosuppressed, especially among patients who may be unwilling to self-report the condition.

HSV-associated hepatitis has been reported previously, but diagnosis of HSV hepatitis is complicated by the lack of specific clinical indications [13–15]. Early reports focused on HSV-related liver failure in neonates and children, and the patient described in the first report of HSV hepatitis in adults was a pregnant woman with several exacerbating factors, including hyperemesis gravidarum and treatment with tetracycline and stelazine [14]. In a more recent study of 5 cases of HSV-related ALF, 4 of the patients were immunosuppressed, and three of the patients were superinfected with HSV1 or HBV [15]. Three patients underwent liver transplantation. Two patients died within three days of admission, and the remaining patients died within a year after transplantation. Each of the patients presented with fever but without skin lesions, and HSV was not initially suspected. The most similar case is a recent report of HSV-associated ALF in an immunocompetent adult following a tongue piercing who presented with worsening fever and inflammation around the piercing site [13]. The patient died while in preparation for liver transplantation. HSV infection was detected during postmortem examination. These diverse cases have few features in common other than fever but together highlight the difficulty of diagnosis as well as the high mortality and rapid progression of HSV-associated ALF.

The case presented here also shows the potential for next generation sequencing technologies in diagnosis of cases of ALF of unclear etiology. Although the etiologies of many cases of ALF remain indeterminate, analysis of stored blood or tissue samples may retrospectively help to identify the cause, which may in turn help to improve diagnosis and treatment of ALF in future cases. In the simplest case, direct sequencing using PCR primers can be used to identify suspected viral pathogens. Next generation sequencing using mapping against a reference panel can also be used to identify suspected as well as unsuspected pathogens, and de novo assembly could be used to identify novel viruses following subtraction of host reads [18].

Accurate identification of the etiology using unbiased methods is important to establish and confirm an association between risk factors and clinical outcome. Early reports implicated viruses such as TTV and GBV-C as potential causes of hepatitis, but later studies have failed to identify an association between the presence of these viruses and acute hepatitis and ALF [19–21]. However, it remains possible that other viruses have yet to be identified. Retrospective sequence analysis will help to establish the role of these and other viruses in the etiology of ALF. Sequence data can also be mapped against the human genome and used to measure differential gene expression relative to healthy controls [22]. Microarrays could also be used for this purpose and compared against publically available expression data [23, 24]. Establishment of gene expression profiles from patients with ALF of known etiology might reveal useful biomarkers that could facilitate diagnosis and lead to more effective treatment.

While the sequencing and mapping approach described here is neither appropriate nor intended for diagnosis of patients in real time, given the urgency of decisions that must be made following admission as well as the costs and time required for sample preparation and analysis. However, the speed and affordability of next generation sequencing platforms is continuously improving, and systems such as the MinION Nanopore sequencer and its competitors are designed to support low-cost, real-time, long read benchtop sequencing of viral and plasmid genomes to rapidly identify viral pathogens or detect antibiotic resistance genes in clinical isolates [10, 11]. There is an urgent need for such systems to be able to respond effectively during outbreaks. By exchanging a measure of accuracy for a much faster turnaround time, cutting edge sequencing tools claim to achieve adequate sequencing results within 20 minutes with a laboratory prep time of only 10 minutes.

Even when sequencing results are already available, sequence identification using BLAST or mapping to a panel of reference genomes is a time-consuming and computationally demanding step, especially when the sample contains mainly human DNA or RNA. However, again by sacrificing accuracy for speed, kmer-based tools such as Taxonomer are able to perform rapid taxonomic profiling of reads using a web-based interface in real time [25]. The software begins classifying the reads while the raw fastq sequencing files are still uploading and can report a rough taxonomic classification down to genus or even species level within several minutes based on a random sample of reads. The combination of rapid sequencing methods with real-time classification methods sets the stage for a new era of informed diagnosis, although it is unclear when these methods will become routinely used in clinical practice.

Although the patient described here recovered without liver transplantation, the outcome might have been different, and had HSV infection been considered as a cause, effective antiviral treatment could have been started promptly. By reevaluating the etiology of unsolved ALF cases using next generation sequencing, we hope to identify overlooked causes in order to improve treatment for future patients. Next generation sequencing provides an unbiased method to detect agents not diagnosed at presentation and might also be useful in identifying unknown viruses.

## Figures and Tables

**Table 1:** Laboratory data at time of admission.

Hematologic test	
White blood cells (/*μ*L)	3740
Neutrophils (%)	80
Lymphocytes (%)	6
Monocytes (%)	4
Eosinophils (%)	0
Basophils (%)	0
Red blood cells (×10^4^/*μ*L)	3.12
Hemoglobin (g/dL)	9.3
Platelet count (×10^4^/*μ*L)	1.1
Coagulation	
Prothrombin time (%)	17
Prothrombin time-INR	2.92
Chemistry	
Aspartate aminotransferase (IU/L)	8676
Alanine aminotransferase (IU/L)	5114
Total bilirubin (mg/dL)	4.3
Direct bilirubin (mg/dL)	2.7
Alkaline phosphatase (IU/L)	480
Lactate dehydrogenase (IU/L)	9120
*γ*-Glutamyltranspeptidase (IU/L)	206
Blood urea nitrogen (mg/dL)	6
Creatinine (mg/dL)	0.5
C-reactive protein (mg/dL)	3.06
Procalcitonin (ng/mL)	0.15
Total protein (g/dL)	5.3
Albumin (g/dL)	2.7
Sodium (mmol/L)	136
Chloride (mmol/L)	92
Potassium (mmol/L)	3.4
Ferritin (ng/mL)	34780
Copper (*μ*g/dL)	53
Ceruloplasmin (mg/dL)	19
Ammonia (*μ*mol/L)	17
IgG (mg/dL)	1028
IgM (mg/dL)	210
IgA (mg/dL)	206
Anti-nuclear antibodies (<)	×80
Hepatitis B surface antigen (IU/mL)	0.02
Hepatitis B core antibodies (COI)	0.1
Hepatitis C virus antibodies (COI)	0.1
IgM-hepatitis A virus antibodies	<0.4
Epstein-Barr virus	
Anti-VCA IgG	80
Anti-VCA IgM	<10
Anti-VCA IgA	<10
Anti-EBNA antibodies	40
Cytomegalovirus	
IgG (−)	26
IgM (−)	(−)
HIV-1,2 antibodies	(−)

COI: cut-off index; INR: international normalized ratio.

## Abbreviations

ALF: Acute liver failure
ALT: Alanine aminotransferase
AST: Aspartate aminotransferase
CT: Computed tomography
HSV: Herpes simplex virus
LT: Liver transplantation.
